# Factors associated with unfavorable evolution of tuberculosis in HIV infected persons

**DOI:** 10.1186/1471-2334-13-S1-P16

**Published:** 2013-12-16

**Authors:** Florentina Dumitrescu, Andreea Cristina Stoian, Augustin Cupşa, Mihai Jianu, Alina Lungu, Alexandra Corobea, Dina Maria Cupşa, Adrian Neguleț

**Affiliations:** 1 University of Medicine and Pharmacy Craiova, Romania; 2 “Victor Babeş” Clinical Hospital of Infectious Diseases and Pneumology, Craiova, Romania

## Background

Tuberculosis (TB) is one of the most common opportunistic infections in HIV-infected patients, with severe evolutive potential.

We evaluated the prevalence and the clinical aspects of TB in HIV-infected persons in order to identify factors associated with unfavorable evolution of TB.

## Methods

We performed an observational, retrospective study (01 January 2009 – 31 December 2011) on 387 HIV-infected patients in evidence at the HIV/AIDS Craiova Regional Center. We analyzed the epidemiological, clinical and paraclinical data (smears and cultures for *Mycobacterium tuberculosis*, immunovirological evaluation) for patients who presented at least 2 times per year at the regional center.

## Results

During the studied period 59 patients (15.3%) presented TB (9 cases simultaneously diagnosed with HIV and TB). General data on the study group: average age at the moment of TB diagnosis: 25.5±7.4 years; equal gender distribution: male/female 30/29; rural/urban 40/19 (67.8/32.2%); average CD4 count 3 months before TB diagnosis: 179±204 cells/cmm; average viral load: 5.09±5.3 log_10_. TB was: pulmonary: 46 cases (77.9%), extrapulmonary 8 (13.6%), multiple locations 5 (8.5%). 19 cases (32.2%) were bacteriologically confirmed (positive smears and/or positive cultures). 53 patients (89.8%) were under antiretroviral treatment, with a very good adherence in 19 patients (32.2%). Other opportunistic infections apart from TB were recorded in 28 patients (47.4%). 29 patients (49.2%) had favorable outcome, complication or relapse occurred in 10 cases (16.9%) and 20 patients (33.9%) died. Factors associated with unfavorable evolution were: delay in the introduction of anti-TB treatment more than 30 days from the first symptom (p=0.001); the presence of other opportunistic infections apart from TB (p=0.0001), low adherence to treatment (p=0.0001). Death was associated with extrapulmonary/multiple location of TB (p=0.005) and average CD4<100 cells/cmm (p=0.002).

## Conclusion

Tuberculosis is common in patients infected with HIV, the unfavorable evolution being associated with severe immunosuppression, extrapulmonary TB location, poor adherence and delay in the introduction of anti-TB treatment.

